# Hydrological pulse regulating the bacterial heterotrophic metabolism between Amazonian mainstems and floodplain lakes

**DOI:** 10.3389/fmicb.2015.01054

**Published:** 2015-09-30

**Authors:** Luciana O. Vidal, Gwenäel Abril, Luiz F. Artigas, Michaela L. Melo, Marcelo C. Bernardes, Lúcia M. Lobão, Mariana C. Reis, Patrícia Moreira-Turcq, Marc Benedetti, Valdemar L. Tornisielo, Fabio Roland

**Affiliations:** ^1^Laboratório de Ciências Ambientais, Centro de Biociências e Biotecnologia, Universidade Estadual do Norte FluminenseRio de Janeiro, Brazil; ^2^Laboratoire Environnements et Paléoenvironnements Océaniques, Université Bordeaux 1Bordeaux, France; ^3^Departamento de Geoquímica, Universidade Federal FluminenseRio de Janeiro, Brazil; ^4^Laboratoire d’Océanologie et Géosciences, Université du Littoral Côte d’OpaleWimereux, France; ^5^Departamento de Hidrobiologia, Universidade Federal de São CarlosSão Carlos, Brazil; ^6^Departamento de Biologia, Instituto de Ciências Biológicas, Universidade Federal de Juiz de ForaJuiz de Fora, Brazil; ^7^Institut de Recherche pour le Développement, Géosciences Environnement ToulouseLima, Peru; ^8^Institut de Physique du Globe de Paris, Sorbonne Paris Cité, Université Paris DiderotParis, France; ^9^Laboratório de Ecotoxicologia, Centro de Energia Nuclear e Agricultura, São Paulo Universidade Federal de São PauloSão Paulo, Brazil

**Keywords:** bacterial production, bacterial respiration, bacterial carbon demand, bacterial growth efficiency, hydrological pulse, Amazonian freshwater ecosystems

## Abstract

We evaluated *in situ* rates of bacterial carbon processing in Amazonian floodplain lakes and mainstems, during both high water (HW) and low water (LW) phases (*p* < 0.05). Our results showed that bacterial production (BP) was lower and more variable than bacterial respiration, determined as total respiration. Bacterial carbon demand was mostly accounted by BR and presented the same pattern that BR in both water phases. Bacterial growth efficiency (BGE) showed a wide range (0.2–23%) and low mean value of 3 and 6%, (in HW and LW, respectively) suggesting that dissolved organic carbon was mostly allocated to catabolic metabolism. However, BGE was regulated by BP in LW phase. Consequently, changes in BGE showed the same pattern that BP. In addition, the hydrological pulse effects on mainstems and floodplains lakes connectivity were found for BP and BGE in LW. Multiple correlation analyses revealed that indexes of organic matter (OM) quality (chlorophyll-a, N stable isotopes and C/N ratios) were the strongest seasonal drivers of bacterial carbon metabolism. Our work indicated that: (i) the bacterial metabolism was mostly driven by respiration in Amazonian aquatic ecosystems resulting in low BGE in either high or LW phase; (ii) the hydrological pulse regulated the bacterial heterotrophic metabolism between Amazonian mainstems and floodplain lakes mostly driven by OM quality.

## Introduction

Bacterioplankton (generic term including heterotrophic aerobic Bacteria and Archaea) is considered the main agent for the removal of organic carbon (C) in aquatic systems (Williams, 2000). Bacterioplankton convert dissolved organic carbon (DOC) into biomass through bacterial production (BP) and into CO_2_ through bacterial respiration (BR). The whole amount of carbon consumed by the bacterial metabolism on BP and BR has been referred as bacterial uptake or bacterial carbon demand (BCD), an important pathway in the global carbon cycle. The efficiency of bacterial carbon assimilation will determine if carbon is either passed to the next trophic level or converted into CO_2_ (Del Giorgio et al., 1999). Bacterial communities in tropical inland aquatic ecosystems have higher metabolic rates (BP, BR, and BCD) and lower bacterial growth efficiency (BGE) than in temperate ecosystems (Amado et al., 2013).

In particular, BR measurements require the separation of bacteria from the rest of the plankton community. The filtration process can cause: the removal of the pressure by competitors and predators (Martínez-García et al., 2013); structure disruption of bacterial community (Del Giorgio and Cole, 1998; Del Giorgio et al., 2011); phytoplankton cells to suffer rupture; and the release of labile organic C to the filtered (Hopkinson et al., 1989) resulting overestimation of respiration measurements. On the other hand, the filtration process may cause an underestimation when particles are retained, which causes a significant percentage of adhered bacteria. Bacteria within a water column can reach high abundances in microhabitats such as aggregates (Grossart and Tang, 2010). This aspect is enhanced in Amazonian freshwater ecosystems due to the high levels of suspended materials. Amazonian aquatic ecosystems show high levels of turbidity reaching 151 nephelometric turbidity units (NTU) in mainstems and 128 NTU in floodplains lakes.

Large parts of the Amazon River are subjected to periodical floods in the surrounding central Amazon area, due mainly to rainfall in the headwaters (Junk, 1997). This creates large temporary wetlands called floodplain, which account with rivers for a total area of ca. 350,000 km^2^ (Melack and Hess, 2011). The periodical connectivity of the rivers creates a great diversity of carbon sources across the mainstems and floodplains lakes (Mortillaro et al., 2011). The organic matter (OM) has been reported as refractory in the mainstem and it has been described as more labile in the Amazonian floodplains (Hedges et al., 1986; Moreira-Turcq et al., 2003; Aufdenkampe et al., 2007). Dissolved organic matter (DOM) originating from aquatic primary producers (planktonic algae and aquatic macrophytes) is usually more labile to bacterial growth (Amon and Benner, 1996; Farjalla et al., 2006). Composition and quality of OM in the Amazon Basin have been previously documented using stable isotopes, chlorophyll-a, elemental analysis, fatty acids (FAs), amino acids, and lignin phenols (Hedges et al., 1994; Bernardes et al., 2004; Mortillaro et al., 2011; Moreira-Turcq et al., 2013). In turn, these factors are important regulators of bacterial activity in aquatic systems (Del Giorgio and Cole, 1998) and can have effects on metabolic efficiency (i.e., BGE) under different environmental conditions (Hall and Cotner, 2007).

The aim of this study was to evaluate the changes in the BP, BR, BGE, and BCD in floodplain/mainstem Amazonian ecosystems.

## Materials and Methods

### Study Site

**FIGURE 1:**
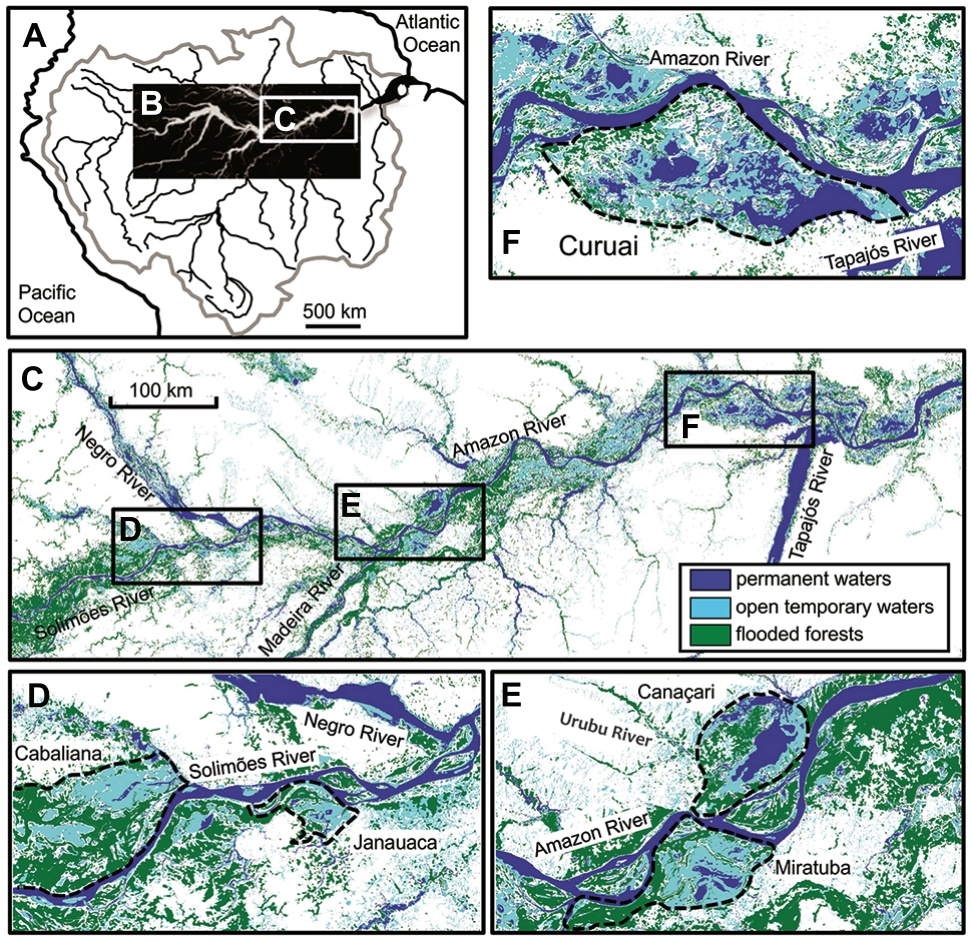
**Location of study areas across the Amazonian River ecosystems in a large **(A–C)** and small scale **(D–F)**.** Dashed black lines delineate the Cabaliana, Janauaca, Miratuba, Canaçari, and Curuai floodplains (Abril et al., 2014).

The Amazon River is the world’s largest river with a drainage basin area of 6.1 × 10^6^ km^2^ covering about 40% of South America (Goulding et al., 2003). The mean annual discharge is 200 × 10^3^ m^3^ s^-1^ at Óbidos, the most downstream gauging station in the Amazon River (Callede et al., 2000). Due to the equatorial position (**Figures 1A–C**), temperature in the central Amazon basin is relatively constant around the year with a mean annual air temperature (MAAT) of ca. 27°C (New et al., 2002). Rivers within the Amazon drainage basin are traditionally classified according to water color, as well as physical and chemical parameters (Sioli, 1956): white water (e.g., Solimões and Madeira), black water (e.g., Negro), and clear water (e.g., Tapajós). Large parts of the central Amazon basin are subjected to periodical floods due mainly to spatial and temporal distribution of rainfall in the headwaters. This creates large temporary wetlands, i.e., seasonally flooded forests which cover a total area of ca. 350 × 10^3^ km^2^ (Junk, 1997; Melack and Hess, 2011). There is water exchange between flooded forests and the Amazon River which is highly influenced by the rising and falling of water during the rainy or dry seasons, respectively (Lesack and Melack, 1995). Five floodplain lakes were investigated in this study (**Figure 1C**): Cabaliana, Janauaca, Miritituba, Canaçari, and Curuai (**Figures 1D–F**), located in a gradient of decreasing flooded forests and increasing open waters (Bourgoin et al., 2007). Cabaliana has a large area surrounded by flooded forests and two sub-regions. In the northern region, Piranha stream discharges black water while in the southern region white water, brought by the Solimões River, mixes with black water. Janauaca has a peculiar morphology with a ravine shape surrounded by flooded forests. Solimões water comes through the channel in the north and black water comes through the stream system in the south. Miritituba receives white water from the Madeira River and the Amazon River. It can be considered as a white water lake surrounded by flooded forests, with no significant contribution of black water streams. Canaçari has two well-defined sub-regions. In the northern region, the Urubu River discharges black water and in the southern region, the Amazon River discharges white water. Canaçari is surrounded by grass plains but is disconnected from flooded forests most of the year. Curuai is the largest lake in the central Amazon basin, mainly surrounded by grass plains and open waters. It receives white water from the Amazon River through small channels, apart from the main channel in the eastern side. There are no significant contributions of black water streams for Curuai Lake.

### Sampling

The main channels of five mainstems were selected (Solimões, Negro, Madeira, Amazon, and Tapajós; **Figure 1**) as well as five floodplain lakes (Cabaliana, Janauacá, Canaçari, Miratuba, and Curuai; **Figure 1**). In total, three to six subsurface water samples were collected at the mainstem stations, and six to seven samples were collected at the floodplain lake stations. Samples were collected from an 800 km transect along the lower Amazon River basin from Manacapuru on the Solimões River to Santarém at the mouth of the Tapajós River. Two cruises were conducted, one in June 2009 during the high water (HW) season and October 2009, 1 month before the lowest water stage, referred to here as the low water (LW) season. In June, as the water level was the highest recorded in the last century, the study area was extensively inundated, enhancing exchange and mixing between the river mainstem, the flooded forest and the open floodplain lakes. In October, the water level was minimal, allowing little interaction with the main channel. The difference in water level at Óbidos between HW and LW was 6 m. The amplitude is generally 3–4 m upstream Manaus (Sioli, 1984).

Subsurface waters (1 m) were collected with a Van Dorn sampler from the ship on seven lakes in HW and LW. Subsamples for chlorophyll a, Tot-P, pH, turbidity, DOC, ^13^δC of the particulate organic carbon (POC), conductivity, O_2_ and bacterial abundance (BA) were taken immediately after sampling. The samples for bacterial parameters were always collected between 7:00 and 11:00 a.m. into acid-washed plastic bottles.

### Bacterial Analytical Methods

Bacterial production was measured in unfiltered samples right after sampling using the 4,5-^3^H L-leucine [specific activity (SA), 50 Ci mmol^-1^] incorporation based on the method of cold trichloroacetic acid (TCA) extraction (Simon and Azam, 1989; Wetzel and Likens, 2000). Ten millilitre of the sample were placed in a vial and leucine added at final concentration of 50 nM. Three replicate tubes plus two blanks were incubated in the dark for 30 min. After, the TCA was added to a final concentration of 20% and then incubated at 4°C. In addition, the samples were filtered through 0.22 μm Nuclepore membranes (Thomaz and Wetzel, 1995), being washed with TCA 5% and ethanol. The leucine incorporated into the bacterial biomass was measured in a Beckman LS 6500 scintillation counter. The protein production was converted to carbon production using a protein-to-carbon ratio of 0.86 (Wetzel and Likens, 2000).

Bacterial respiration was measured by following changes in dissolved oxygen during dark incubations. Boro-silicate glass bottles were carefully filled and three replicates were immediately fixed with Winkler reagents to determine the initial oxygen concentration. Three replicate bottles were incubated in the dark at *in situ* temperature and fixed with Winkler reagents after12 h. Dissolved oxygen measurements were made with an automatic titrator (DL50 Graphix, Mettler Toledo) based on potentiometric endpoint detection (Granéli and Granéli, 1991). The respiration rate was determined by the difference between final and initial samples measured at times zero and 12 h. To convert mg O_2_ L^-1^ to mg C L^-1^, we used a respiration quotient (RQ) = 1. In order to detect possible interferences of filtration on BR measurements, tests were conducted in June 2009 (HW) on filtered and unfiltered samples (total sample) from 13 stations. The BR was measured by following changes in dissolved oxygen during dark incubations of total and filtered water following the procedures described above. The filtered fraction was obtained from total samples filtered under low pressure with a vacuum pump through 1.2 μm (GF/C) Whatmann fiber filters right after sampling. The respiration measurements on the filtered fractions were significantly higher than the unfiltered samples (*p* < 0.05) for 100% of the stations (1.6 times higher). Therefore, the measurement obtained from the unfiltered water, and not the filtered fraction, was used as the estimate of BR in the present study.

Bacterioplankton abundance was estimated on subsamples collected in 40 ml sterile polyethylene flasks, preserved in borate-buffered 0.2 μm pre-filtered formalin (3% final concentration), and stored at 4°C. Subsamples were stained with DAPI (4 μg mL^-1^) for 15 min (Porter and Feig, 1980), filtrated through 0.2 μm black polycarbonate membranes (Millipore© Isopore) previously mounted on GF/C Whatman fiber filters to optimize cell distribution, then mounted on slides with non-fluorescent oil (Olympus optical). Direct counts were performed at 1,250× magnification under an epifluorescence microscope (Leica Leitz DMR; 365 nm). In high-turbid waters, subsamples were pre-treated (before staining) by an addition of 150 μL of Tween, sonicated at 35 kHz for 5 min, and centrifuged at 3,000 *g* during 10 min at 4°C (Chevaldonné and Godfroy, 1997; Hubas et al., 2007). The abundance of free bacteria was estimated in untreated diluted samples and/or in subsamples pre-filtered through a 3-μm filter. The attached bacteria were deduced from total to free bacterial counts.

### Bacterial Carbon Fluxes

Bacterial growth efficiency was calculated as BP/BCD (Del Giorgio and Cole, 1998). BCD was defined as the sum of BR and BP. BR, BP, and BCD were expressed as μg C L^-1^ h^-1^.

### Ancillary Parameters

Water temperature, conductivity, pH, O_2_, and turbidity were measured with an YSI^®^ multiprobe, calibrated every 10 days. Position was recorded with a global positioning system (GPS) at the same frequency of 1 min. During LW periods, most shallow and remote lakes were visited with the aid of a small boat and a 12V version of the complete measurement setup was used. DOC concentration was measured on water that was filtered through pre-combusted GF/F filters (4 h, 550°C), stored in pre-combusted glass bottles and acidified with ultrapure H_3_PO_4_. DOC concentrations were measured using a Shimadzu TOC-V_CSH_ analyzer. The detection limit for carbon was 4 μg/L. POC, total nitrogen (TN, i.e., organic and inorganic nitrogen), δ^13^C and δ^15^N isotope ratios were measured on the same sample aliquots by EA-IRMS (Carlo-Erba NA-1500 NC Elemental Analyser on line with a Fisons Optima Isotope Ratio Mass Spectrometer). The δ^13^C and δ^15^N of POC values are reported in per mil (‰) relative to Pee Dee Belemnite (PDB) standard and relative to air N_2_, respectively. The analytical precision (as the standard deviation of repeated internal standard measurements) for the stable isotope measurements was 0.06 and 0.13% for δ^13^C and δ^15^N, respectively (Moreira-Turcq et al., 2013). Chlorophyll-a concentrations (μg L^-1^) were measured on GF/F filters, stored frozen before analysis, according to the method described by Yentsch and Menzel (1963), Holm-Hansen et al. (1965), and (Marker et al., 1980), using a 10-AU Turner Fluorometer.

### Data Analyses

The differences between HW and LW were tested by Mann–Whitney Rank Sum Test because the data did not meet the normality and equal variance test criteria. Potential correlations between variables were determined through Spearman’s correlation analysis without log-transformation of data. All statistical calculations were performed using SigmaPlot v12.5. For all statistical tests we assumed *p* < 0.05 as a threshold level for acceptance.

## Results

### Mainstem and Floodplain Environmental Variability

The subsurface water temperature did not differ between mainstems and floodplain lakes, although in the LW period the temperature was slightly higher (about 2°C) than in HW (**Table 1**). The DOC concentration range was similar in mainstems and floodplain lakes in HW and LW. The POC isotopic signal differed only in mainstems in HW and LW. The chlorophyll-a range was similar in mainstems in HW and LW and increased in floodplain lakes in LW. The average BA was similar between mainstems and floodplain lakes in HW and in mainstems in HW and LW and increased in floodplain lakes in LW.

**Table 1:** Location and general features of the Amazon River subsystem (mainstem and floodplain lakes).

	Mainstem			Floodplain lakes		
	Min.	Max.	Mean (±*SD*)	Min.	Max.	Mean (±*SD*)
**HW**					
Water temperature	27.9	29.9	28.6 (± 0.02)	28.1	29.7	28.85 (± 0.02)
DOC	3.6	7.6	4.3 (± 0.43)	3.6	5.1	4.04 (± 0.15)
^13^C-POC	–27.8	–34.55	30.21 (± 0.07)	–28.1	–30	29.18 (± 0.02)
C/N	6.19	11.43	8.25 (± 0.20)	6.87	8.86	8.15 (± 0.09)
pCO_2_	3650	6850	4555.5 (± 0.43)	3000	8360	5448.5 (± 37)
Turbidity	3,5	151	41.83 (± 1.35)	9.5	61.2	28.9 (± 0.66)
Conductivity	12	56	31.33 (± 0.60)	43	74	49.7 (± 0.25)
pH	4,9	6.4	5.91 (± 0.11)	6.3	6.6	6.4 (± 0.02)
Chlorophyll-a	0.5	3.0	1.7 (± 0.92)	0.9	2.5	1.82 (± 0.40)
0_2_	36.1	71.8	58.9 (± 0.39)	30.9	79.9	51.6 (± 0.34)
BA	0.8	1.3	1.06 (± 0.27)	0.3	1.4	0.97 (± 0.49)
**LW**					
Water Temperature	30.8	31.9	31.2 (± 0.02)	29.5	32.3	30.82 (± 0.04)
DOC	1.4	6.1	4.13 (± 0.59)	3.8	5.5	4.54 (± 0.18)
^13^C-POC	–28	–29.5	28.9 (± 0.02)	–27.1	–29.7	28.46 (± 0.04)
C/N	8.65	11	9.82 (± 0.12)	5.82	8.28	6.68 (± 0.14)
pCO_2_	750	4548	3032.7 (± 0.66)	298	7900	2985 (± 1.95)
Turbidity	6.4	56	23.7 (± 1.81)	17	128	59.6 (± 0.80)
Condensation	8	59	28.3 (± 0.95)	41	78	56.2 (± 0.26)
pH	4.7	6.9	6.1 (± 0.20)	6.6	7.7	7.1 (± 0.06)
Chlorophyll-a	1.7	9.8	4.83 (± 0.90)	9.3	73.7	32.74 (± 0.78)
0_2_	73	100.4	83.9 (± 0.17)	63	110	86.4 (± 0.25)
BA	0.3	1.2	0.63 (± 0.78)	0.9	2.9	1.17 (± 0.29)

*Water temperature (°C); dissolved organic carbon (DOC, mg L-^1^); stable isotope signal of POC (^13^C-POC, ‰); partial pressure of the CO_2_ (pCO_2_, μatm); turbidity (NTU); conductivity (m s^-1^); pH; Chlorophyll-a (μg L^-1^); Oxygen (%) and bacterial abundance (BA; 10^6^ cell mL^-1^) and correspond to the respective year of study [High water (HW) and Low water (LW) phases] at each station.*

### BP and BR

**FIGURE 2:**
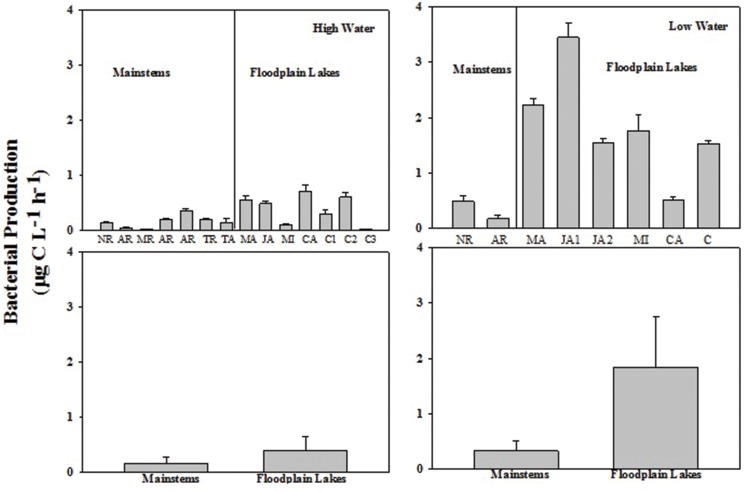
**Bacterial production (BP; μg C L^-1^ h^-1^) by H^3^-Leu incorporation across the Amazon River system in high water (HW; left upper panel) and low water (LW; right upper panel).** CA, Cabaliana; JA, Janauacá; NR, Negro River; AR, Amazonas River; MR, Madeira River; MI, Miratuba; C, Curuai; TA, Tapajós River. Average and standard deviation for the mainstems and floodplain lakes in HW (left bottom panel) and LW (right bottom panel).

**FIGURE 3:**
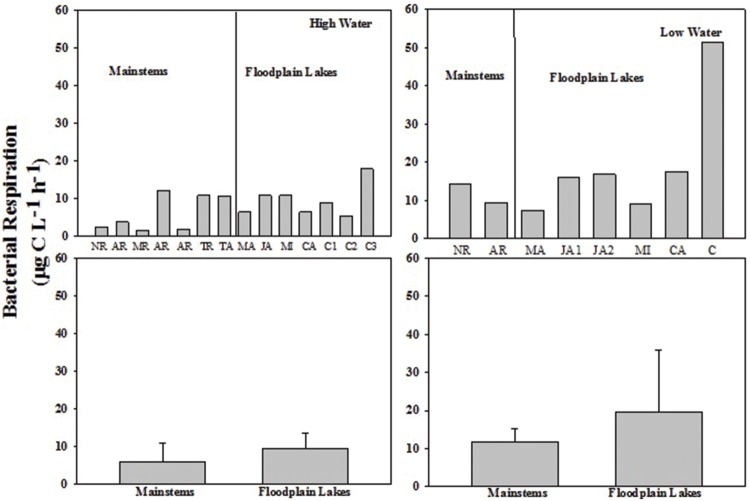
**Bacterial respiration (BR; μg C L^-1^ h^-1^) across the Amazon River system in HW (left upper panel) and LW (right upper panel).** CA, Cabaliana; JA, Janauacá; NR, Negro River; RAR, Amazonas River; MR, Madeira River; MI, Miratuba; C, Curuai; TA, Tapajós River. Average and standard deviation for the mainstems and floodplain lakes in HW (left bottom panel) and LW (right bottom panel).

The BP rates varied from 0.02 to 0.36 μg C L^-1^ h^-1^ (0.16 ± 0.11) in the mainstems and from 0.02 to 0.60 μg C L^-1^ h^-1^ (0.36 ± 0.25) in the floodplain lakes in HW phase. BP ranged from 0.17 to 0.48 μg C L^-1^ h^-1^ (0.33 ± 0.22) in the mainstems and from 0.54 to 3.44 μg C L^-1^ h^-1^ (1.84 ± 0.96) in the floodplain lakes in LW (**Figure 2**). The levels of BP on floodplain lakes were significantly higher (*p* < 0.05) than on mainstems in LW. No difference was found between mainstems and floodplain lakes on HW. The BR rates varied from 1.54 to 12.15 μg C L^-1^ h^-1^ (6.11 ± 4.80) in the mainstems and from 5.43 to 17.82 μg C L^-1^ h^-1^ (9.41 ± 4.30) on floodplain lakes in HW (**Figure 3**). The levels of BR in LW varied from 9.43 to 14.28 μg C L^-1^ h^-1^ (11.85 ± 3.42) on mainstems and from 7.34 to 51.5 μg C L^-1^ h^-1^ (19.70 ± 16.16) on floodplain lakes. The BR did not show any significant difference between mainstems and floodplain lakes in HW and LW (*p* > 0.05).

### BCD and BGE

**FIGURE 4:**
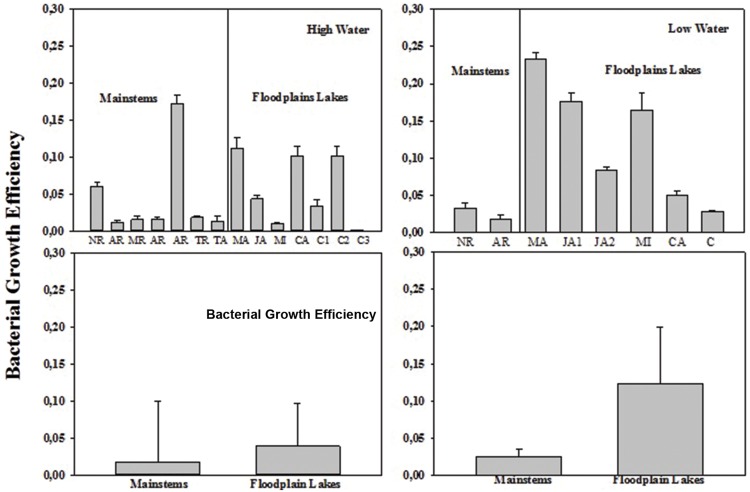
**Bacterial growth efficiency (BGE) across the Amazon River system in HW (left upper panel) and LW (right upper panel).** CA, Cabaliana; JA, Janauacá; NR, Negro River; AR, Amazonas River; MR, Madeira River; MI, Miratuba; C, Curuai; TA, Tapajós River (upper panel). Average and standard deviation for the mainstems and floodplain lakes in HW (left bottom panel) and LW (right bottom panel).

**FIGURE 5:**
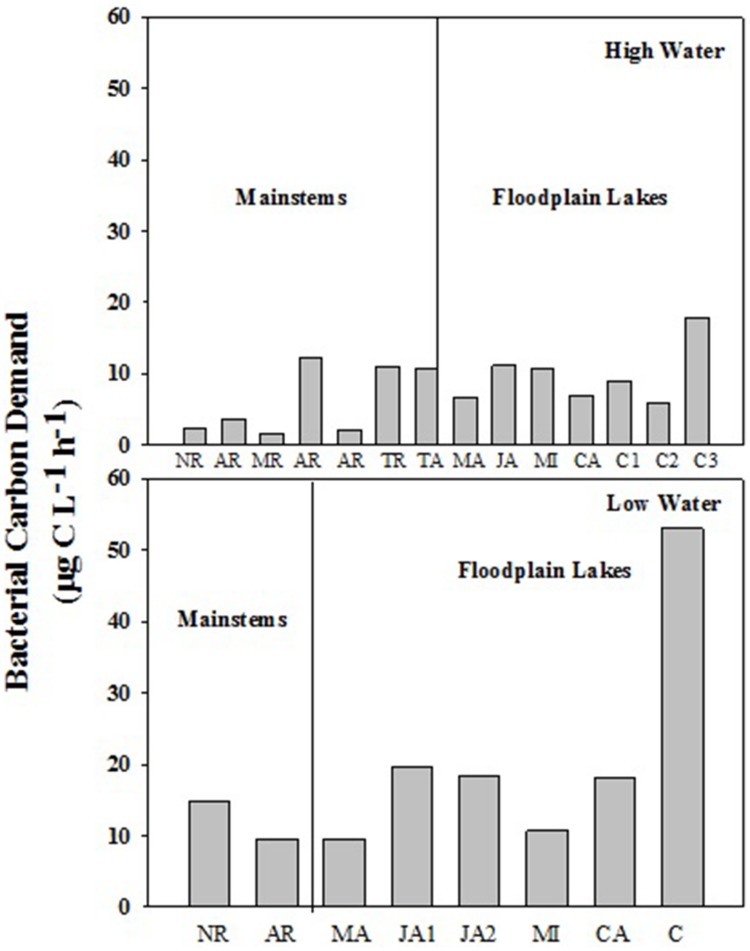
**Bacterial carbon demand (BCD) across the Amazon River system in HW (left upper panel) and LW (right upper panel).** CA, Cabaliana; JA, Janauacá; NR, Negro River; AR, Amazonas River; MR, Madeira River; MI, Miratuba; C, Curuai; TA, Tapajós River (upper panel). Average and standard deviation for the mainstems and floodplain lakes in HW (left bottom panel) and LW (right bottom panel).

The BCD varied from 1.58 to 12.36 μg C L^-1^ h^-1^ (6.27 ± 4.82) in mainstems and from 5.94 to 17.84 μg C L^-1^ h^-1^ (8.88 ± 5.33) in floodplain lakes in HW (**Figure 4**). In LW, BCD varied from 9.6 to 14.76 μg C L^-1^ h^-1^ (12.18 ± 3.65) in mainstems and from 9.57 to 19.62 μg C L^-1^ h^-1^ (15.98 ± 4.57) in floodplain lakes. The BCD did not show any significant difference between mainstems and floodplain lakes in HW and LW (*p* > 0.05). The lack of correlation between the simultaneous measurements of BP and BR in both sampling conditions resulted in a wide range of calculated BGE. BGE varied from 0.01 to 0.18 (0.05 ± 0.06) on mainstems and from 0.002 to 0.07 (0.05 ± 0.03) on floodplain lakes in HW, and from 0.02 to 0.03 (0.02 ± 0.007) in mainstems and from 0.03 to 0.23 (0.12 ± 0.08) in floodplain lakes in LW (**Figure 5**). The BGE in floodplain lakes were significantly higher (*p* < 0.05) than in mainstem in LW (**Figure 4**) and mainly driven by BP (**Tables 2** and **3**). During HWs, no significant difference was observed between the mainstem and the floodplain lakes (*p* > 0.05). No correlation was found between BP and BR in either sampling periods.

**Table 2:** Spearman correlation coeficients between measured variables in Amazonian systems to HW period; ^∗^*p* < 0.05, ^∗∗^*p* < 0.01, ^∗∗∗^*p* < 0.0001.

	BR	BP	BCD	BGE	CHLOR-A	TURB	BA	DOC	C13	C/N	δ^15^N
BR	–										
BP	ns	–									
BCD	0.98^∗∗∗^	ns	–								
BGE	ns	0.85^∗∗∗^	ns	–							
CHLOR-A	ns	ns	ns	ns	–						
TURB	ns	ns	ns	ns	–0.68^∗∗^	–					
BA	0.50^∗^	ns	ns	ns	ns	ns	–				
DOC	ns	ns	ns	ns	ns	ns	ns	–			
C13	ns	ns	ns	ns	–0.53^∗∗^	0.81^∗∗∗^	ns	ns	–		
C/N	ns	ns	ns	0.54^∗∗^	ns	–0.64^∗∗^	ns	ns	ns	–	
δ^15^N	ns	ns	ns	ns	ns	ns	ns	ns	ns	ns	–

*BR, bacterial respiration; BP, bacterial production; BCD, bacterial carbon demand; BGE, bacterial growth efficiency; CHLOR-A, chlorophyll-a; TURB, turbidity; BA, bacterial abundance; DOC, dissolved organic carbon; POC, stable isotopic of POC; C/N, carbon and nitrogen ratio; δ^15^N, stable isotopic of nitrogen.*

**Table 3:** Spearman correlation coeficients between measured variables in Amazonian systems to LW period; ^∗^*p* < 0.05, ^∗∗^*p* < 0.01, ^∗∗∗^*p* < 0.0001.

	BR	BP	BCD	BGE	CHLOR-A	TURB	BA	DOC	C13	C/N	δ^15^N
BR	–										
BP	ns	–									
BCD	0.88^∗∗^	ns	–								
BGE	ns	0.93^∗∗^	ns	–							
CHLOR-A	ns	ns	0.68^∗∗^	ns	–						
TURB	ns	ns	ns	ns	ns	–					
BA	ns	ns	ns	ns	0.93^∗∗∗^	ns	–				
DOC	ns	ns	ns	ns	ns	ns	ns	–			
C13	ns	ns	ns	ns	ns	ns	ns	ns	–		
C/N	ns	ns	ns	ns	–0.89^∗∗^	ns	ns	0.77^∗∗^	ns	–	
δ^15^N	ns	–0.77^∗∗^	ns	–0.75^∗∗^	ns	ns	ns	ns	ns	ns	–

*BR, bacterial respiration; BP, bacterial production; BCD, bacterial carbon demand; BGE, bacterial growth efficiency; CHLOR-A, chlorophyll-a; TURB, turbidity; BA, bacterial abundance; DOC, dissolved organic carbon; C13, stable isotopic of POC; C/N, carbon and nitrogen ratio; δ^15^N, stable isotopic of nitrogen.*

### Bacterial Metabolism Correlation with Environmental Factors

Indexes of OM quality (chlorophyll-a, δ^15^N, and C/N ratios) were the strongest seasonal statistical predictors of bacterial carbon metabolism in the Amazonian subsurface waters. BP, BR, BCD, and BGE were not correlated to DOC or C^13^ (**Tables 2** and **3**). In contrast, the POC OM stoichiometry (C/N) showed a significant negative correlation to BGE (*r* = –0.54, *p* < 0.05) in HW. Besides, there was a strong tendency of increasing BA and BCD with chlorophyll-a concentration in LW (*r* = 0.93, *p* < 0.0001; *r* = 0.68, *p* < 0.05). The δ^15^N of POC presented strong negative correlation to BP and BGE (*r* = -0.77 and –0.75, *p* < 0.05) in LW.

## Discussion

The assessment of bacterial metabolic rates (BR, BP, BCD, and BGE) from Amazonian freshwater ecosystems is poorly understood. Most of studies considered only one or few freshwater ecosystems (Farjalla et al., 2002, 2006; Castillo et al., 2003; Amado et al., 2006). A study conducted by Benner et al. (1995) was the most comprehensive study considering samples taken from mainstems and tributaries. It showed that bacterial metabolism, represented by BP and BR, presented minimal spatial variability in Amazonian tributaries and mainstems but strong seasonal patterns of variability. Rivers are very dynamic and potentially subject to great spatial heterogeneity, making the identification of regulatory factors on bacterial community highly complex. Such heterogeneity suggests that a dynamic and variable microbial metabolism might be expected in rivers (Del Giorgio et al., 2011). Recently, Farjalla (2014) showed that BP increases in areas of mixing zones of Amazonian rivers. The Amazonian mainstems and floodplains studied in the present study reflect the wide variability of the world’s largest river system (**Table 1**).

The consumption of OM by bacteria is driven by OM amount and composition (Kritzberg et al., 2005; Jansson et al., 2006; Vidal et al., 2011), therefore, the range of metabolic versatility of heterotrophic bacterial metabolism has often been assumed to occur as the response to large shifts in resources across major ecosystems or along extremely broad environmental gradients (Del Giorgio et al., 2011).

In Amazonian freshwater ecosystems, the carbon from the forest goes into the floodplain lakes and meets the in-lake OM production (i.e., phytoplankton and macrophytes), which is part processed before reaching the mainstem channel (Moreira-Turcq et al., 2003). Besides, the percentage of forest occupying the drainage basin in the study area showed a well-defined biogeographic gradient from flooded forests that are dominant upstream to open lakes and that are dominant downstream with temporal scale across the floodplain lakes (Abril et al., 2014). Consequently, the forest carbon litterfall entering the aquatic ecosystems may be also variable. The spatial variability of organic carbon across the Amazon floodplain basin where OM is spatially and seasonally structured may facilitates the evolution of a broad repertoire of functional attributes of bacteria to their environment (Comte and del Giorgio, 2010). In addition, floodplains represent a hotspot of primary production during LW and, consequently, a source of presumably fresh suspended particulate organic matter (SPOM) for much of the system, particularly from macrophytes. Seasonal water movements are a way to redistribute this fresh SPOM in the hydrological network via the transfer to the river main channel (Mortillaro et al., 2012).

Bacterial metabolism in Amazonian aquatic ecosystems is inversely related to the water level. The phytoplankton and macrophytes are the main forms of organic carbon at LW phase, and the allochthonous input of OM is a significant energy source to bacterial activity during HW level (Amado et al., 2006). It is also known that the synergy between low- and high-molecular-weight carbon sources and consequences for the bacterioplankton has recently been pointed out (Fonte et al., 2013). For instance, recent findings suggest that lignin and other terrestrially derived macromolecules contribute significantly to carbon dioxide outgassing from inland waters thorough microbial degradation in Amazon River systems (Ward et al., 2013). In the present study, it was possible to notice a clear difference on bacterial metabolism through BP and BGE between HW and LW phases and also between mainstems and floodplain lakes in LW (**Figures 2** and **4**). The range of bacterial metabolic processes found in the Amazonian freshwater ecosystems showed to be in part driven by the diversity of OM available across the Amazonian floodplain basin. Our results showed that indexes of OM quality (chlorophyll-a, N stable isotopes, and C/N ratios) were the strongest seasonal drivers of bacterial carbon metabolism. The effects of OM fate (origin and decomposition stage) on bacterial carbon consumption in the present study were suggested by the negative correlation of BGE and BP with C:N ratio in HW and by the strong negative coupling with δ^15^N in LW. The increasing in stoichiometry of carbon may indicate carbon of terrestrial origin and N limitation, characteristic of periods of HW when the carbon from the forest is dominating and the nutrients are more diluted in the water. The higher δ^15^N values attest for enhanced nitrogen recycling in the system (Ometto et al., 2006) in LW when there is an increasing in carbon from phytoplankton origin and nutrients availability. Indeed, positive relationships were evidenced between phytoplankton (expressed as chlorophyll-a concentration) and heterotrophic bacteria (expressed as abundance) and BCD in LW. The DOC quantity did not account for variations in BP and BR in the present study on both sampling times. Such lack of relationship between DOC and bacterial metabolism parameters may indicate that not the carbon concentration but the carbon quality is accounting for such variations. Recent studies in boreal and Amazonian freshwater ecosystems have shown that C alone is not enough to regulate bacterial metabolism; but instead, the stoichiometry of carbon must be considered. (Farjalla et al., 2002; Castillo et al., 2003; Vidal et al., 2011).

The BP rates were the responsible for most of BGE variability (Roland and Cole, 1999; Kritzberg et al., 2005) but not enough to increase BGE according to the present study. The higher respiration rates in relation to production showed that most of the BCD is converted into CO_2_, which resulted in low BGE values in both sampling phases, (0.03 in HW and 0.06 in LW). These higher metabolic rates (e.g., BCD) and lower BGE values in tropical inland water ecosystems are lower than those observed in temperate ecosystems which is related to temperature when inserted in a global scenario, but also to intrinsic ecosystems aspects (Amado et al., 2013) like OM quality, as indicated in this study. Moreover, regarding BR rates, it is important to keep in mind that in the unfiltered water used during respiration incubations a range of protists and metazoans must have contributed directly to heterotrophic respiration resulting in BR and BCD overestimations and consequently BGE underestimations. In addition, trophic interactions may also have affected respiration rates as suggested by Biddanda et al. (2001), with bacteria accounting less with total respiration with increasing in system productivity. However, the respiration measurements between high and LW phase did not show any significant difference in the present study, which may indicate that increasing in phytoplankton production showed through chlorophyll-a concentration in LW, did not resulted in changes in respiration rates. This was reinforced by the no correlation found between BR and Chlorophyll-a (**Tables 2** and **3**). Thus, we believe such possible bias of the measurements with no filtration would not affect the seasonal pattern found in the present study.

## Concluding Remarks

Our results showed that the hydrologic connectivity, through carbon quality (chlorophyll-a, N stable isotopes, and C/N ratios), differentially drives bacterial carbon allocation across freshwater Amazonian ecosystems. In general, it showed higher BP in LW phase than in HW phase and higher BR rates than BP in both water phases. In addition, BR rates did not show any significant difference between HW and LW (*p* > 0.05). The same was registered to BCD. Average BGE was low in both seasons (0.03 and 0.06, HW and LW, respectively), suggesting that DOC was mostly allocated to catabolic bacterial cell processes besides BP increasing in LW. Consequently, changes in BP and BGE between the deep rivers and the adjacent shallow lakes showed the same pattern as BP. Multiple correlation analyses revealed that indexes of OM quality (chlorophyll-a, DOC and DOC, N stable isotopes, and C/N ratios) were the strongest seasonal drivers of bacterial carbon metabolism.

Our work indicated that: (i) the bacterial metabolism was mostly driven by respiration in Amazonian aquatic ecosystems resulting in low BGE in either HW or LW phase; (ii) the hydrological pulse regulated the bacterial heterotrophic metabolism between Amazonian mainstems and floodplain lakes, mostly driven by OM quality.

From the results presented in this study we could increase the discussion about bacterial carbon metabolism in Amazon floodplain ecosystems and we tried to fill the gap about *in situ* detailed knowledge of local factors regulating microbial metabolism.

## Conflict of Interest Statement

The authors declare that the research was conducted in the absence of any commercial or financial relationships that could be construed as a potential conflict of interest.
